# Cardiovascular Benefits of Menopause Hormone Treatment is Age-Dependent

**DOI:** 10.64898/2026.06.29.734522

**Published:** 2026-07-01

**Authors:** Cassandra D. Lalisan, Connor Gavin, Bridget Litts, Jeffrey A. Rein, Julia An, Min Zhong, Rajguru Boobalan, Yu Wang, Amelia Van Aelst, Sivaprakasam Chinnarasu, Yaomin Xu, MacRae Linton, John M. Stafford, Lin Zhu

**Affiliations:** 1 Division of Endocrinology, Diabetes, and Metabolism, Department of Internal Medicine, The Ohio State University Medical Center, Columbus, OH.; 2 Tennessee Valley Healthcare System, Nashville, TN.; 3 Division of Diabetes, Metabolism and Endocrinology, Vanderbilt University Medical Center, Nashville, TN.; 4 Division of Cardiology, Department of Medicine, Vanderbilt University Medical Center, Nashville, TN.; 5 Department of Molecular Physiology and Biophysics, Vanderbilt University, Nashville, TN.; 6 Department of Biostatistics, Vanderbilt University, Nashville, TN.; 7 Department of Health Data Science and Biostatistics, Peter O’Donnell Jr. School of Public Health, UT Southwestern Medical Center, Dallas, TX.

## Abstract

**Background:**

Hormone therapy (HT) has not consistently reduced atherosclerotic cardiovascular disease (ASCVD) events in post-menopausal women, yet the underlying mechanisms remain poorly understood.

**Methods:**

Female *Ldlr*^−/−^ mice with established atherosclerosis were subjected to surgical menopause and treated with 17β-estradiol (E_2_) following lipid normalization. Studies were performed in aging and young mice. To determine whether inflammation mediates the age-dependent response to HT, a cohort of aging mice underwent transplantation with *Ifnγ*^−/−^ bone marrow (BM) before hormone treatments. Metabolic parameters, HDL function, systemic inflammation, atherosclerotic burden, liver metabolic and oxidative stress signaling, and hepatic estrogen receptor signaling were evaluated.

**Results:**

In aging mice, menopause E_2_ treatment failed to reduce established atherosclerosis as shown in sham operated mice during lipid normalization. Instead, E_2_ treatment increased circulating IFNγ and IL-6, impaired HDL antioxidant and cholesterol efflux functions, and promoted inflammatory and vulnerable plaque phenotypes. Suppression of inflammation through *Ifnγ*^−/−*BM*^ transplantation restored HDL function and significantly reduced atherosclerosis in E_2_-treated aging mice. In contrast to aging mice, young mice exhibited reduced systemic and plaque inflammation, improved HDL functions and atherosclerosis following E_2_ treatment. Liver RNA sequencing and qPCR validation identified activation of inflammatory, oxidative stress, and lipid metabolic pathways in aging E_2_-treated mice, which were largely attenuated following *Ifnγ*^−/−^ bone marrow transplantation as well as in young mice. Compared to young mice, aging mice presented hepatic estrogen receptor remodeling characterized by reduced estrogen receptor α (ERα) expression and increased G-protein coupled estrogen receptor (GPER) expression. Constitutive GPER activation was accompanied by induction of NOX1-dependent oxidative stress, which was further exacerbated by E_2_ treatment, leading to persistent inflammation.

**Conclusions:**

The cardiovascular effects of estrogen therapy are fundamentally age dependent. Aging shifts estrogen signaling toward hepatic oxidative stress and inflammation through increased GPER. While E_2_ treatment preserves both metabolic and cardiovascular protection in young mice, aging exacerbates GPER-NOX1-mediated oxidative stress, resulting in impaired HDL function and persistent residual ASCVD risk. These findings identify inflammation-driven, non-lipid mechanisms as potential therapeutic targets to improve cardiovascular outcomes during hormone therapy in postmenopausal women.

## Introduction

Atherosclerotic cardiovascular diseases (ASCVD) is a leading cause of mortality worldwide and the leading cause of death for women in the United States. Pre-menopausal women have about half the risk of developing atherosclerosis compared with men of the same age^1,2^ and typically experience ASCVD onsets nearly a decade later. This woman-specific cardiometabolic protection is largely lost after menopause as circulating estrogen levels decline, suggesting estrogens play a key role in the cardiometabolic benefits in premenopausal women. However, hormone therapy (HT) approaches have not consistently replicated this kind of protection in postmenopausal women.

The cardiometabolic benefits of estrogens has been demonstrated in clinical and pre-clinical studies. Estrogens play a critical role in maintaining metabolic health in both women and men. In women who undergo bilateral oophorectomy before age 45, HT improves HDL cholesterol levels and attenuates many of the adverse metabolic changes that occur after surgery^3^. In men, estrogens are primarily generated through the conversion of testosterone and androstenedione to estradiol and estrone, respectively, by the enzyme aromatase^4^. Men with aromatase deficiency or mutations in the ERα gene often present central adiposity, insulin resistance, fatty liver disease, and impaired glucose homeostasis, all of which can be improved by HT^4–6^. Consistent with these observations, experimental suppression of aromatase activity in healthy men induces insulin resistance, highlighting the essential role of estrogens in regulating metabolism and insulin sensitivity^7^.

Mechanisms underlying the metabolic benefits of estrogens have been extensively elucidated in preclinical studies. Estrogens exert their biological effects through three receptors: ERα, ERβ, and GPER. Among these, ERα is the principal mediator of metabolic homeostasis, playing a critical role in maintaining glucose homeostasis^8,9^. In skeletal muscle, estrogens enhance insulin sensitivity through multiple actions, including ERα-mediated Akt activation, which promotes glucose transporter type 4 (GLUT4) translocation and glucose uptake^10,11^. Estrogens stimulate mitochondrial biogenesis through phosphoinositide 3-kinase (PI3K) and extracellular signal-related kinase 1 and 2 (ERK1/2) signaling pathways^12^. Increased mitochondrial capacity supports efficient fatty acid oxidation and prevents the accumulation of lipid intermediates that impair insulin signaling. In adipose tissue, estrogens promote adiponectin secretion and ERα-dependent browning of white adipose tissue, thereby enhancing energy expenditure^13^. We have previously demonstrated that hepatic ERα signaling is required to limit liver fat accumulation and maintaining glucose homeostasis during high fat diet feeding^14,15^. Interestingly, estrogen treatment improved metabolism but induced pro-inflammatory effects in ovariectomized rats fed a high fat diet, highlighting a potential mechanistic link between estrogen's beneficial metabolic actions and its context-dependent adverse cardiovascular effects^16^.

Recent meta-analyses of clinical trials show that HT, including both mono-estrogen and combined estrogen-progestin regimens, improves glucose homeostasis and related metabolic parameters in non-diabetic postmenopausal women^17^. However, regarding ASCVD events, HT approaches have not yet shown consistent benefits in postmenopausal women^18–20^. Cardiovascular benefits observed in early years of HT led to the “timing hypothesis,” which posits that HT effects depend on the timing of initiation relative to age and years since menopause^21^. Support for this concept is primarily from The Early versus Late Intervention Trial with Estradiol (ELITE), which enrolled healthy post-menopausal women without diabetes or clinical evidence of atherosclerosis to receive placebo or oral estradiol^22^. Participants were stratified according to menopause onset. Women who initiated HT within 6 years of menopause exhibited significantly slower progression of carotid intima-media thickness (CIMT), a surrogate marker of atherosclerosis, compared with placebo-treated controls after 5 years of intervention^22^. In contrast, among women who initiated HT more than 10 years after menopause, no significant difference in CIMT progression was observed between the HT and placebo groups^22^.

In the Heart and Estrogen/Progestin Replacement Studies and follow up prognostic studies (HERS/HERS II), post-menopausal women with established coronary atherosclerotic disease were enrolled^18,19^. Despite favorable changes in lipid profiles, HT did not significantly reduce the overall incidence of primary outcomes, including myocardial infarction and coronary heart disease death, nor did it improve their secondary outcomes such as cardiac arrest, stroke, and transient ischemic attack after ~7 years follow up^18,19^. Similar findings were reported in other trials involving postmenopausal women with clinical evidence of pre-existing atherosclerosis, including the Estrogen Replacement and Atherosclerosis Trial (ERA), Women’s Estrogen-Progestin Lipid-Lowering Hormone Atherosclerosis Regression Trial (WELL-HART), and Women’s Angiographic Vitamin and Estrogen Trial (WAVE) studies^20,21,23–25^. Notably, increases in inflammatory markers, such as C-reactive protein, were observed following HT, suggesting that persistent inflammation may diminish the cardiovascular benefits of hormone therapy^20,23^.

To understand the underlying mechanisms for the discordance between metabolic improvements of hormone therapy and its limited cardiovascular benefits, we developed mouse models of preexisting atherosclerosis incorporating 17β-estradiol (E_2_) treatment after surgical menopause (ovariectomy) across the lifespan of hyperlipidemia mice. Our findings show that E_2_ treatment in aging *Ldlr*^−/−^ mice with established atherosclerosis improves metabolic parameters but increases systemic inflammation and fails to reduce atherosclerotic burden during hyperlipidemia normalization. Importantly, suppression of inflammation through expanding *Ifnγ*^−/−^ bone marrow significantly attenuates atherosclerosis in aging mice during menopausal E_2_ treatment. These findings closely mirror clinical observations and support a critical role for inflammation-driven, non-lipid mechanisms in residual ASCVD risk during hormone therapy. Mechanistically, the persistent inflammatory state in aging E_2_-treated mice is associated with increased expression of genes in inflammatory and hepatic oxidative stress pathways. In contrast, young mice exhibit markedly lower inflammatory and oxidative stress responses to E_2_ treatment, and reduced atherosclerotic burden. The expression of inflammatory genes is estrogen responsive in both aging and young mice. Additionally, we observed age-dependent ER remodeling in the liver, characterized by reduced hepatic ERα dominance and enrichment of ERβ and GPER signaling in aging mice. Constitutive activation of GPER induces NADPH oxidase 1 (NOX1), leading to increased oxidative stress and inflammatory signaling. Our findings provide mechanistic insight into how aging-driven dysfunction and estrogen-induced inflammation may compromise the cardiometabolic benefits of hormone therapy and contribute to residual ASCVD risk in postmenopausal women.

## Materials and Methods

### Animal Studies:

The mouse colonies are housed and maintained in 12-hour light/dark cycles in temperature and humidity-controlled facilities with ad-libitum access to a chow and water. *Ldlr*^−/−^ (Jax. Strain #002207) and *Ifnγ*^−/−^ (Jax. Strain #00287) mice were originally purchased from Jax lab. All mouse experiments were approved under the Institutional Animal Care and Use Committee at Vanderbilt University Medical Center and The Ohio State University.

**Figure 1.**
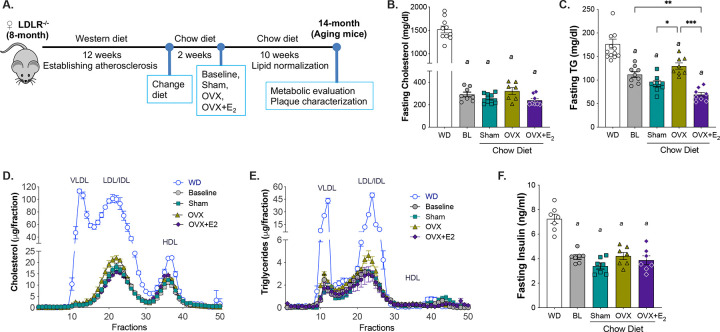
Estrogen treatment improves metabolic parameters following lipid normalization in aging *Ldlr*^−/−^ mice. **A.** Schematic of the study design. **B-C:** Fasting serum total cholesterol levels. (B)Fasting serum triglyceride (TG) levels (C). **D-E**: Serum cholesterol (D) and TG (E) distribution determined by fast protein liquid chromatography (FPLC). **F.** Fasting serum insulin concentrations. Data are presented as mean ± SEM (n >=7). Statistical analyses were performed using one-way ANOVA. *a*: *P*<0.05 compared to Western diet (WD) conditions. *, *P* < 0.05; **, *P* < 0.01; ***, *P* < 0.001.

Cardiometabolic effects of hormone treatments: To investigate inflammation with hormone therapy in postmenopausal women with preexisting atherosclerosis, we developed mouse models including established atherosclerosis, normalization of hyperlipidemia, and menopause E_2_ treatment (Fig. 1A). Briefly, atherosclerosis is established with Western diet (Research Diets Inc., D12079B) in female *Ldlr*^−/−^ mice. 12 weeks later, WD was changed to a chow diet. A group of mice were sacrificed 14 days after changing diet to chow when atherosclerosis peaks and served as baseline control. The rest of the mice underwent surgeries for hormone treatments, i.e. E_2_ treatment after surgical menopause by ovariectomy (OVX+E_2_), OVX, and sham, following the procedures we reported before^14,26^. Immediately following ovariectomy, E_2_ treatment was administered through the same incision to subcutaneously implant a β estradiol pellet (Innovative Research, NE 121, 0.25 mg, 90 day release) in the shoulder region using sterile forceps. Post operative analgesia was administered after surgery with repeat doses every 12hrs for at least 48hrs, or longer as needed based on pain assessment. After an additional 10 weeks of chow diet feeding, mice were sacrificed after 5 hours of fasting; serum, liver tissues, and aorta were collected and appropriately stored for the analysis of metabolic changes and atherosclerosis burden.

Bone Marrow Transplant (BMT): In this study, inflammation was suppressed by bone marrow transplantation using donor cells from *Ifnγ*^−/−^ mice as we reported before^27^. Briefly, after 12-week of WD feeding, mice were reconstituted with *Ifnγ*^−/−^ bone marrow from 6–8 weeks old donor mice following fetal irradiation (900 rad). All mice were recovered on a chow diet for 2 weeks and subjected to surgeries for hormone treatments^14^.

### Lipid, insulin, and inflammatory marker assessments

Fast protein liquid chromatography (FPLC, Superose 6 column, GE healthcare) was utilized to separate lipoproteins (VLDL, LDL, HDL) from pooled serum samples from 2–3 mice. Cholesterol and triglyceride levels in total serum and FPLC fractions were assessed via enzymatic colorimetric assays using colorimetric kits (Cholesterol Reagent and Triglycerides GPO Reagent kits, Raichem, San Diego, CA) as we described before^28^.

Fasting serum insulin concentrations were measured using a commercially available mouse insulin ELISA kit (Crystal Chem, Cat. # 90080) according to the manufacturer’s instructions. Circulating inflammatory markers in aging mice, including recipients of *Ifnγ*^−/−*BM*^ transplantation, were quantified using mouse IFNγ (R&D Systems, Cat. # MIF00) and IL-6 (R&D Systems, Cat. # M600B) ELISA kits according to the manufacturer’s protocols.

Liver lipid contents for all mice and circulating inflammatory markers for young mice were assessed by the Vanderbilt Medical Center Hormone & Analytical Service Core^29^.

### HDL function assays

HDL function was evaluated with ApoB-free serum as we reported before^30^.

Antioxidant capacity was quantified using the OxiSelect Total Antioxidant Capacity Assay Kit (Cell Biolabs, Cat. # STA-360) according to the manufacturer’s instructions. Total antioxidant power was converted and represented as Copper Reducing Equivalents (CRE) as we described before^31^.

Cholesterol efflux assays were performed following the protocol we reported before. Briefly, human monocyte THP-1 cells were differentiated into macrophages using phorbol 12-myristate 13-acetate (PMA, 10ng/mL, Sigma-Aldrich) treatment for 48hr. The PMA-treated THP-1 cells were then washed twice with PBS and seeded onto 24-well plates (3×10^5^ cells per well). 4 hours later, non-adherent cells are removed, and plates were washed twice with PBS. Cells were incubated with a mixture of 3μci/mL 1,2-^3^H-cholesterol and acetylated LDL (250 μg/mL, Alfa Aesar) in DMEM with 0.25% BSA (Sigma Aldrich, MAK192) or were labelled with fluorescent cholesterol in a Labeling Reagent (Abcam, ab196985) for 1hr at 37°C. Cells were cultured in DMEM with 0.25% BSA and ^3^H-cholesterol+LDL or Equilibration Buffer + RPMI at 37°C overnight. Cells are then washed twice with PBS and then treated with HDL diluted in 0.25% BSA in DMEM or treated with HDL diluted in RPMI and incubated for 4hr at 37°C. Cells were then filtered out (Millipore Cat No. MAHVN4510) for media collection that was analyzed using Liquid scintillation counting (LSC) or cells were lysed (Lysis Buffer I, Abcam) and lysate was measured on a spectral microplate reader (SpectraMax M2) at 523nm. Cholesterol efflux capacity was calculated by dividing measured fluorescence intensity in the supernatant by the sum of the fluorescence intensity of the supernatant and cell lysate, then multiplied by 100.

### Characterization of atherosclerotic lesion

We characterized atherosclerotic burden by analyzing atherosclerotic lesion sizes, lesion inflammation and vulnerability. Lesion sizes were quantified after frozen cross sections of aortic root were stained with Oil Red O as we previously described^26,30,32^ . Lesion inflammation was evaluated with CD68^+^ staining. Briefly, sections were fixed in cold acetone for 10min, washed twice with PBS, blocked in background buster (Innovex) for an 1hr at 37°C, and incubated with primary antibody for CD68^+^ (Calbiochem) at 4°C overnight. After incubation, sections were washed 3 times with PBS and incubated with secondary antibodies (Alexa Fluor 488 anti-rabbit IgG, Life Technologies) at 37°C for 1hr. Lesion vulnerability was analyzed by necrotic core quantification as previously described^32^. Images were captured using an Olympus IX81 microscope and analyzed using the KS300 imaging system (Kontron Elektronik GmbH) or Image J software.

### Liver Bulk RNAseq and Gene Expression

Liver mRNA was assessed through bulk-RNAseq and qPCR for gene expression changes. Mice were sacrificed, and liver samples were flash-frozen in liquid nitrogen immediately and stored in −80°C until later use. Liver tissue (25mg) was bead-homogenized and total RNA was isolated according to manufacturer’s instructions (Direct-zol RNA Miniprep Kit, Zymo Research, USA). Isolated RNA was treated with DNAse I and submitted (~80ng/μL) to Vanderbilt Technologies for Advanced Genomics (VANTAGE) core laboratory for Next-Generation sequencing. RNA samples were sequenced using multiplexed Paired-End 150bp on the Illumina NovaSeq 6000, quality control (QC) was evaluated at different levels including RNA quality, raw read data, alignment, and gene expression. Raw paired-end reads were mapped to the mouse reference genome mm10 using STAR 2.7.3^33^. Feature count was used to calculate raw read counts and downstream analysis^34^. Differential gene expression and functional enrichment analyses were performed in R (version 3.6) with several Bioconductor packages: DESeq2, clusterProfiler. Comparisons were made between the sham and E_2_-treatment groups. Results for specific comparison were extracted by combinations of coefficients with DESeq2. A 5% false discovery rate threshold was used for significant results filtering.

Gene expression was validated with qPCR. RNAs were isolated from liver samples and complementary DNA was synthesized from 1μg of RNA (iScript, Bio-Rad, USA). Quantitative PCR was performed in duplicate using TaqMan primers (Supplemental Table 1) and reagents.

### Statistical Analysis

Data are expressed as mean±SEM unless otherwise stated. Data are summarized using the mean and standard error of the mean. Statistical differences were analyzed by proper ANOVA with post-hoc multiple comparison test as indicated in each figure legend. P-values <0.05 were considered statistically significant. Statistical analysis of RNA-seq datasets is indicated above.

## Results

### Menopause hormone treatment fails to improve atherosclerosis following lipid normalization in aging mice.

To model postmenopausal hormone therapy in the setting of established atherosclerosis, female *Ldlr*^−/−^ mice were fed a Western diet for 12 weeks beginning at 8 months of age to induce atherosclerosis and were subsequently switched to a chow diet to normalize plasma lipids (Fig. 1A). Two weeks after switching diet, when the atherosclerotic plaque peaks, one group of mice were euthanized for baseline plaque evaluation. The rest of the mice were divided into three groups with matching body composition for hormone treatments, including sham surgery, ovariectomy (OVX), and OVX with estradiol replacement (OVX+E_2_). After 10 weeks of treatments while fed a chow diet, mice were euthanized for the assessment of metabolic parameters and atherosclerotic burden. Chow diet significantly reduced fasting triglyceride (TG) and cholesterol levels, accompanied by markedly improved lipoprotein profiles across all hormone treatment groups. (Fig. 1B). During lipid normalization, fasting TG concentrations remained elevated in OVX mice compared with baseline and sham-operated groups, whereas estrogen treatment with 17β-estradiol (E_2_) significantly reduced triglyceride levels to values below baseline groups (Fig. 1C), and cholesterol levels were not significantly different between hormone treatment groups (Fig. 1D). Lipid normalization markedly decreased atherogenic VLDL- and LDL-associated cholesterol and TGs compared with Western diet-fed mice (Fig. 1D–E and Suppl. Fig. 1). Analysis of lipid distribution revealed that LDL-cholesterol was higher in OVX mice than both sham and OVX+E_2_ mice, whereas TG content within VLDL fractions was in a trend of increase after ovariectomy compared with sham mice, consistent with the reduction in fasting plasma TGs (Suppl. Fig. 1). Fasting insulin concentrations were significantly reduced in all chow-fed groups relative to the Western diet group and were not significantly different between hormone treatment groups (Fig. 1F). These findings demonstrate that switching from Western diet to chow diet effectively normalizes hyperlipidemia and improves insulin homeostasis in aging *Ldlr*^−/−^ mice. Importantly, estrogen treatment further improves triglyceride metabolism during lipid normalization, supporting a beneficial effect of hormone therapy on systemic metabolic function.

**Figure 2.**
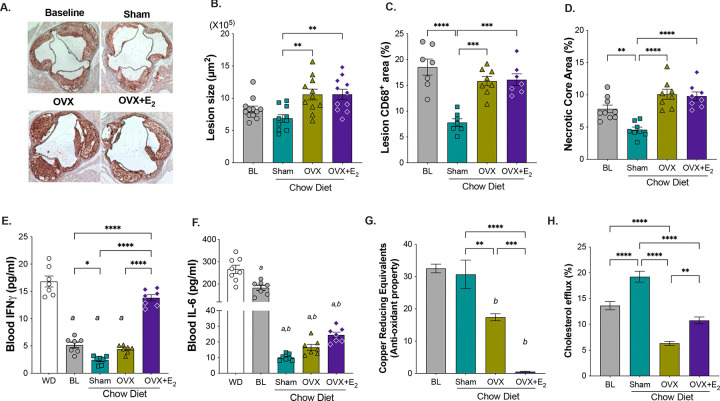
Estrogen treatment fails to reduce atherosclerotic burden during lipid normalization in aging mice. **A.** Representative Oil Red O staining of aortic root sections. **B.** Quantification Oil Red O positive lesion area in the aortic root. **C.** Atherosclerotic plaque inflammatory status assessed by CD68^+^ immunofluorescent staining for macrophage-positive area. **D.** Plaque vulnerability assessed by quantification of necrotic core area. **E-F:** Circulating concentrations of inflammatory markers IFNγ (E) and IL-6 (F) levels. **G-H:** HDL anti-oxidative property (G) and cholesterol efflux capacity (H). Data are presented as mean ± SEM (n>=7). Statistical analysis was performed using one-way ANOVA. *a*: P<0.05 compared to Western Diet (WD) feeding conditions; *b*: *P*<0.05 compared to baseline (BL) conditions; *, *P*<0.05; **, *P*<0.01; ***, *P*<0.001; ****, *P*<0.0001.

To explore the impact of estradiol treatment on atherosclerotic burden, we analyzed lesion area in aortic root through Oil Red O quantification for lesion sizes, CD68^+^ immunofluorescent staining for inflammatory responses, and necrotic core area for plaque vulnerability. The overall lesion sizes of hormone treatment groups were not significantly different from baseline, yet OVX and OVX+E_2_ mice had significantly higher lesion area than sham-operated group (Fig. 2A–B). CD68^+^ area, stained as a marker for macrophages, was significantly higher in OVX and OVX+E_2_ mice than in sham mice (Fig. 2C). Plaque vulnerability represented by necrotic core area demonstrated a similar profile seen in CD68^+^ staining, significantly higher in OVX and OVX+E_2_ mice than in sham mice (Fig. 2D). Plaque vulnerability and inflammation are strongly associated with clinical cardiovascular events in humans. Notably, both vulnerability and inflammation were reduced in sham mice compared with baseline, indicating an improvement in atherosclerotic burden that was not presented in OVX+E_2_ mice.

We next evaluated circulating inflammatory markers and HDL function to identify a potential mechanism for the failure to reduce atherosclerotic burden. Lipid normalization significantly reduced circulating inflammatory markers in baseline as well as in hormone treatment groups; however, circulating IFNγ maintained high levels in OVX+E_2_ mice, about 4 times higher than in sham mice (Fig. 2E). Furthermore, IL-6 levels were also higher in OVX+E_2_ mice than in OVX and sham mice (Fig. 2F). We previously reported increased inflammation compromises HDL function^29^. In line with the persistent high levels of circulating inflammatory markers, anti-atherogenic function of HDL particles from OVX+E_2_ mice were attenuated compared with sham mice. Notably, the antioxidant capacity of HDLs from OVX+E_2_ mice was almost completely diminished, even lower than OVX mice (Fig. 2G). Cholesterol efflux is the mechanism by which HDL particles remove excess cholesterol from blood, beginning the process of reverse cholesterol transport where excess lipids are transported to the liver for disposal^30^. Interestingly, the cholesterol efflux capacity was improved in sham mice during lipid normalization compared with baseline. The cholesterol efflux capacity was lower in OVX+E_2_ mice than sham mice, but higher than OVX (Fig. 2H). These data suggests that menopausal E_2_ treatment fails to reduce established atherosclerotic burden in aging *Ldlr*^−/−^ mice after lipid normalization. Instead, E_2_ promotes a more inflammatory and vulnerable plaque phenotype, accompanied by increased systemic inflammation and impaired HDL function.

### Targeting inflammation improves HDL function and reduces atherosclerosis burden in aging mice during E_2_ treatment.

**Figure 3.**
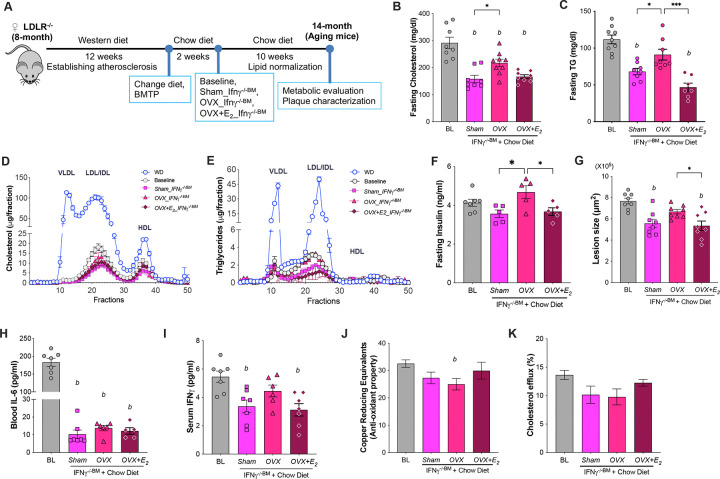
Suppressing inflammation promotes atherosclerosis reversal after lipid normalization in aging female mice during hormone treatment. **A.** Experimental design for suppressing inflammation by *Ifnγ*^−/−^ bone marrow transplant. **B-D:** Fasting serum cholesterol (B), triglyceride (C), and insulin (D) concentrations. **E-F:** Cholesterol (E) and triglyceride (F) lipoprotein distribution was determined by FPLC. **G.** Quantification of aortic root lesion area. **H-I:** Circulating IL-6 (H) and IFNγ (I) concentrations. **J-K:** HDL antioxidant capacity (J) assessed by copper reducing equivalents and HDL cholesterol efflux capacity (K). Data are presented as mean ± SEM. Statistical analyses were performed using one-way ANOVA. *b*: *P*<0.05 compared to baseline (BL) conditions; *, *P*<0.05; ***, *P*<0.001.

We observed failure to confer cardiovascular protection in estrogen-treated ovariectomized mice associated with systemic inflammation. To determine whether persistent inflammation contributes to a lack of cardiovascular benefit, we designed a study to target inflammation directly (Fig. 3A). Similar to our first study, atherosclerosis was established in female *Ldlr*^−/−^ mice through Western diet feed for 12 weeks beginning at 8 months of age. When switching diet to chow, we performed a bone marrow transplant (BMT) and reconstituted the mice with *Ifn*γ^−/−^ bone marrow cells. Two weeks after changing diet to chow, mice underwent hormone treatment procedures and were fed a chow diet for an additional 10 weeks. Consistent with the lipid-lowering effects of chow feeding, fasting serum cholesterol and TG concentrations were significantly reduced in all groups compared with Western diet-fed conditions (data not shown). Unlike our first study, fasting cholesterol and triglyceride levels were further reduced in sham and OVX+E_2_ mice compared with baseline and OVX groups (Fig. 3B–C). These effects likely reflect the contribution of LDLR-expressing cells derived from *Ifn*γ^−/−^ bone marrow following transplantation, together with the established ability of estrogens to upregulate LDLR expression and enhance circulating lipid clearance. Consistent with this observation, lipid content across lipoprotein fractions was lower in bone marrow recipient mice than in the baseline group, with LDL-associated lipids further reduced in sham and OVX+E_2_ mice (Fig. 3D–E, Suppl. Fig. 2). Similarly, fasting insulin concentrations were significantly lower in sham and OVX+E_2_ groups but remained elevated in OVX controls (Fig. 3F). In contrast to our first study, atherosclerotic lesion area was comparably reduced in both sham and OVX+E_2_ groups relative to baseline, whereas lesion burden remained elevated in OVX mice (Fig. 3G). The reduction in atherosclerosis observed in OVX+E_2_ mice was accompanied by lower circulating IL-6 and IFNγ levels (Fig. 3H–I), as well as improved HDL antioxidant activity and cholesterol efflux capacity (Fig. 3J–K). These findings suggest that suppression of inflammatory signaling restores the atheroprotective effects of estrogen treatment during lipid normalization.

### Atheroprotective effects of estrogen treatment may be age dependent.

**Figure 4.**
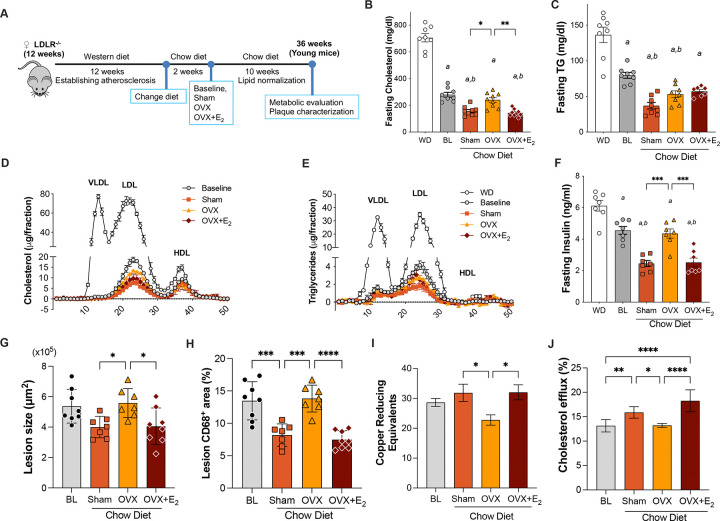
Estrogen treatment reduces plaque burden and HDL function in young *Ldlr*^−/−^ mice following lipid normalization. **A.** Experimental design with younger adult female *Ldlr*^−/−^ mice at the age with reproductive function maintained. **B-C:** Fasting serum cholesterol (B) and triglyceride (C) concentrations were similarly improved in Sham and OVX+E_2_ groups. **D-E:** Lipoprotein distribution of cholesterol (D) and triglyceride (E) was determined by FPLC. **F.** Fasting insulin concentrations were similarly reduced in sham and OVX+E_2_ groups. **G.** Aortic root lesion area was quantified after Oil Red O staining. **H.** CD68^+^ macrophage area within atherosclerotic lesions was quantified after immunofluorescent staining. **I.** HDL cholesterol efflux capacity. **J.** HDL antioxidant capacity measured as copper reducing equivalents. Data are presented as mean ± SEM. Statistical analyses were performed using one-way ANOVA. *a*: P<0.05 compared to Western Diet (WD) feeding conditions; *b*: *P*<0.05 compared to baseline (BL) conditions; *, *P*<0.05; **, *P*<0.01; ***, *P*<0.001; ****, *P*<0.0001.

To determine whether age influences the cardiovascular response to estradiol treatment, we next performed a study without BMT in younger adult mice. In the young mouse model, atherosclerosis was established in 12-week-old *Ldlr*^−/−^ female mice (Fig. 4A). Following hormone treatment procedures and lipid normalization, mice were sacrificed at approximately 8-months of age, a developmental stage where reproductive capacity is still preserved.

As observed in aging mice, transition from Western diet to chow diet substantially reduced plasma lipid levels. Fasting cholesterol and TG concentrations were significantly lower in all chow-fed groups compared with Western diet-fed mice (Fig. 4B–C). Sham mice with intact ovaries and E_2_-treated mice showed further reduced cholesterol levels relative to baseline and OVX groups. Fasting triglyceride concentrations were also further lowered in sham and OVX groups but not in OVX+E_2_ group (Fig. 4C). Consistent with these findings, FPLC analyses demonstrated marked reductions in VLDL- and LDL-associated cholesterol and triglycerides after lipid normalization, with modest improvements in LDL-cholesterol profiles in sham- and E_2_-treated mice compared with baseline and OVX animals (Fig. 4D–E, Suppl. Fig. 3). Fasting insulin levels showed a significant reduction in sham and E_2_-treated mice, a similar improvement as fasting cholesterol (Fig. 4F).

Unlike the findings in aging mice, estradiol treatment promoted regression of established atherosclerotic lesions in young mice. The overall lesion size was not significantly different from baseline; however, sham and E_2_-treated mice had smaller lesion area compared to ovariectomized mice (Fig. 4G). Notably, the baseline lesion area between young and aged mice was substantially smaller in young mice (Figs. 2B and 4G). This finding underscores a common limitation of atherosclerosis regression models, in which relatively small baseline lesions and modest treatment-induced regression reduce the sensitivity for detecting changes in overall lesion size. Regarding atherosclerotic inflammation, sham and estrogen treatment significantly reduced CD68^+^ macrophage content compared with baseline conditions as well as OVX treatment (Fig. 4H), indicating improved plaque resolution of inflammation by estrogens in sham and OVX+E_2_ groups. Importantly, circulating IFNγ and other inflammatory markers were reduced in both sham and OVX+E_2_ groups (Table 1).

Furthermore, HDL function was also strongly influenced by estrogen status. HDL antioxidant capacity was reduced following ovariectomy and restored by estrogen treatment to levels even greater than those observed in OVX mice (Fig. 4I). Similarly, HDL cholesterol efflux capacity was significantly enhanced in both sham and OVX+E_2_ mice compared with baseline and OVX groups (Fig. 4J).

Collectively, these findings demonstrate that estrogen treatment exerts beneficial metabolic and cardiovascular effects in young *Ldlr*^−/−^ mice following lipid normalization. In contrast to aging mice, menopause E_2_ treatment promotes regression of established atherosclerotic lesions, reduces systemic and plaque inflammation, and improves HDL function, indicating that the vascular protective effects of estrogen treatment are preserved in younger animals but become impaired with aging.

### Estrogen treatment induces hepatic metabolic and inflammatory dysregulation in aging mice.

**Figure 5.**
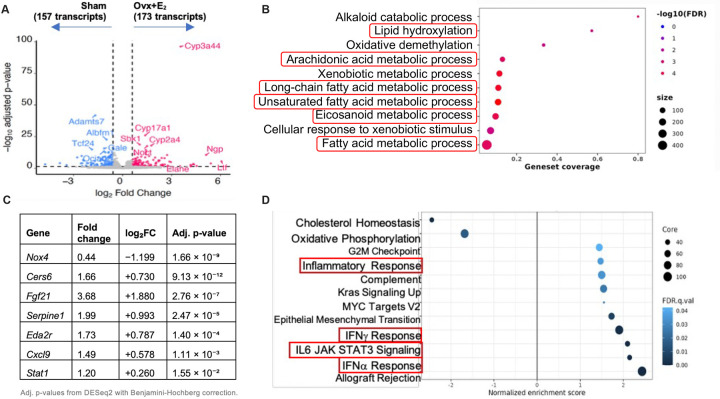
Liver transcriptomic profiling of aging *Ldlr*^−/−^ mice between estrogen treatment and sham procedure. **A.** Volcano plot showing differentially expressed genes in livers from aging OVX+E_2_ mice compared with sham-operated controls. **B.** Six out of top ten significantly enriched biological processes (GO) are lipid metabolism. **C.** Selected differentially expressed genes associated with inflammation, lipid metabolism, oxidative stress, and cellular stress responses. **D.** Four out of twelve significantly enriched pathways (GSEA) are associated with inflammatory signaling.

To investigate potential sources of inflammation in aging mice, we performed bulk RNA-sequencing on sham and E_2_-treated mouse liver tissues. Approximately 330 genes were differentially regulated between the two groups (Fig. 5A), a modest but sufficient change to indicate that E_2_-treatment cannot fully recapitulate the physiological functions of intact ovaries. Among the most strongly induced genes were inflammatory and myeloid-associated transcripts, including *Ltf*, *Ngp*, and *Elane*, suggesting enhanced innate immune activation. Estradiol treatment also increased expression of genes involved in metabolic metabolism and cellular stress responses, including *Cyp17a1*, *Cyp2a4*, and *Sbk1*. Gene ontology analysis revealed that many differentially expressed genes were associated with lipid metabolic pathways (Fig. 5B). Enriched pathways included fatty acid metabolism, long-chain fatty acid metabolism, unsaturated fatty acid metabolism, arachidonic acid metabolism, eicosanoid metabolism, and lipid hydroxylation, indicating substantial remodeling of hepatic lipid handling in response to E_2_ treatment. Representative genes that are involved in those pathways are listed in Fig. 5C. Consistent with these observations, Gene Set Enrichment Analysis (GSEA) demonstrated strong positive enrichment of multiple immune and inflammatory pathways in estradiol-treated mice (Fig. 5D). Hallmark gene sets associated with inflammatory response, IFNγ response, IFNα response, and IL6-JAK-STAT3 signaling were among the most significantly enriched pathways. Complement activation and epithelial-mesenchymal transition pathways were also increased, supporting a pro-inflammatory and tissue-remodeling phenotype. In contrast, pathways related to cholesterol homeostasis and oxidative phosphorylation were negatively enriched, suggesting impaired mitochondrial energy metabolism and disrupted lipid homeostasis.

### The dysregulation of hepatic metabolism and inflammation during menopause E_2_ treatment is age dependent.

**Figure 6.**
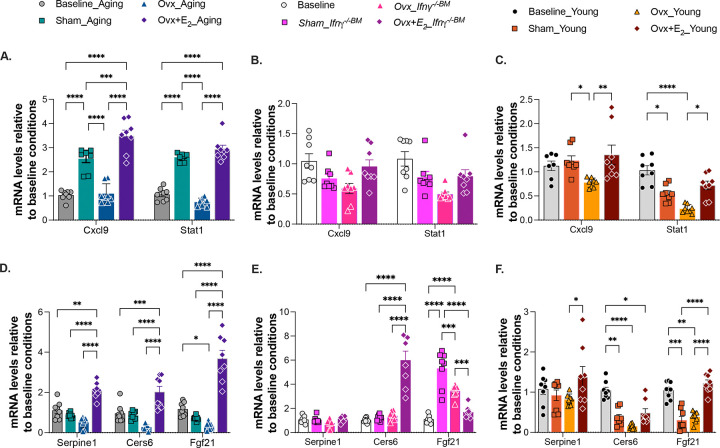
Aging- and estrogen-dependent regulation of hepatic interferon signaling, lipid metabolism, and stress-response genes. **A-C:** Hepatic mRNA expression of interferon-responsive genes in aging mice (A), in aging mice receiving *Ifnγ*^−/−^ bone marrow transplantation (B), and young mice (C). **D-F:** Expression of genes associated with lipid metabolism, cellular stress, and fibrosis in female in aging mice (D), in aging mice receiving *Ifnγ*^−/−^ bone marrow transplantation (E), and in young mice (F). Gene expression was determined normalized to 18s. Data are presented as fold change relative to the corresponding baseline groups. Data are shown as mean ± SEM (n=7–8). Statistical significance was determined by two-way ANOVA with Sidak’s post hoc multiple-comparison tests. *, *P*<0.05, **, P<0.01, ***, *P*<0.001, ****, P<0.0001.

We next validated the expression of genes that are involved in the enriched pathways shown in Fig. 5B and Fig. 5D with liver tissues from mice across ages and treatments. In aging *Ldlr*^−/−^ mice, the expression of IFNγ response genes, *Cxcl9* and *Stat1*, was higher with OVX+E_2_ treatment than sham treatment (Fig. 6A), consistent with bulk-RNAseq analysis. Notably, expression of these genes was also higher in sham treated mice than OVX treated mice and even higher than the baseline condition (Fig. 6A), indicating that expression of these genes is also estrogen responsive and metabolic context dependent. Expression of additional inflammatory genes has been shown in Supplemental Figure 5. Whereas in aging *Ldlr*^−/−^ mice with *Ifnγ*^−/−*BM*^ transplant, induction of inflammatory transcripts was blunted following transplant (Fig. 6B and Suppl. Fig. 5). In young *Ldlr*^−/−^ mice, these transcripts were increased in sham and E_2_-treated mice compared to OVX, supporting the role estrogens play in promoting inflammation; however, the overall pro-inflammatory transcript levels remained comparable or lower to the baseline condition (Fig. 6C).

A similar pattern is observed in genes associated with hepatic stress and metabolic dysfunction. Plasminogen activator inhibitor −1 (PAI-1, gene name: *Serpine1*) is a marker of tissue remodeling and fibrosis; Ceramide synthase 6 (*Cers6*) is a key enzyme involved in ceramide biosynthesis; and fibroblast growth factor 21 (*Fgf21*) is a hepatokine induced by metabolic and cellular stress. Elevated *Cers6* expression suggests increased ceramide synthesis and lipotoxic stress, whereas induction of *Fgf21* is consistent with activation of adaptive metabolic and integrated stress response pathways. Previous studies have shown that *Serpine1* and *Fgf21* are directly regulated by estrogens through ERα binding sites within their promoter regions^35,36^. There is currently no evidence that *Cers6* is a direct ERα target, therefore, its estrogen-responsive expression is likely mediated indirectly through alterations in metabolic pathways downstream of *Fgf21*, *Serpine1*, or other estrogen-regulated signaling pathways.

Although the overall pattern of hormone regulation was preserved in both aging and young mice, the expression of *Serpine1*, *Cers6*, and *Fgf21* was significantly higher in OVX+E_2_ group than in baseline controls only in aging mice, whereas no such induction was observed in young mice (Fig. 6D, F). These findings suggest that estrogen-regulated lipotoxic and cellular stress responses are exacerbated with aging. Notably, the responsiveness of these genes to hormone treatments was markedly attenuated following transplantation of *Ifn*γ^−/−^ bone marrow (Fig. 6E). These results indicate that suppression of inflammation through *Ifn*γ^−/−BM^ transplantation not only reduces hepatic inflammation signaling but also modulates metabolic stress pathways, which is consistent with the altered liver lipid content observed in *Ifn*γ^−/−BM^ recipient mice (Suppl. Fig. 4) and with our previous findings in similar mouse models^27^.

### Age-associated upregulation of hepatic GPER is accompanied by increased oxidative stress and stress-response in the liver from E_2_-treated aging mice.

**Figure 7.**
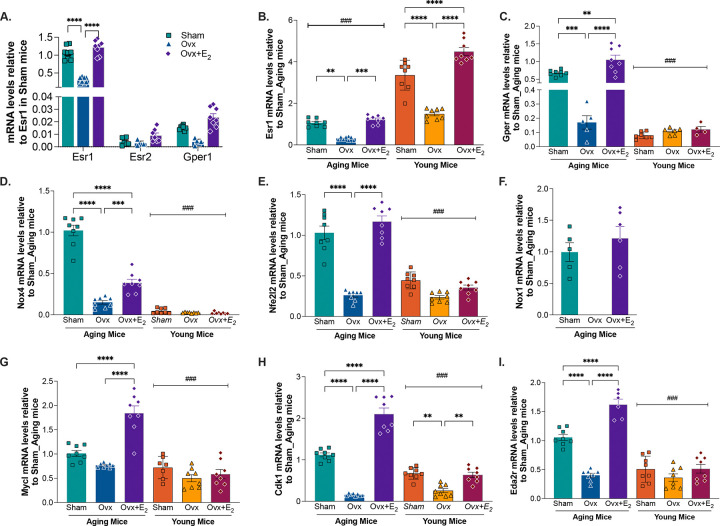
Aging-associated estrogen receptor remodeling and oxidative stress signaling in the liver. **A.** Hepatic mRNA expression of **Esr1**, **Esr2**, and **Gper1** in aging mice. **B-I:** Comparison of hepatic expression of Esr1 (B), Gper1 (C), Nox4 (D), Nfe2l2 (E), Nox1 (F), Mycl1 (G), Cdk1(H), and Eda2r (I) between aging and young mice. mRNAs of Esr2 and Nox1 were not detectable with qPCR in young mice. Gene expression was determined normalized to 18s. Data are presented as fold change relative to the corresponding sham conditions in aging mice for B-I. For panel A, data represented as fold change relative to Esr1 from aging sham mice. Data are shown as mean ± SEM (n=7–8). Statistical significance was determined by two-way ANOVA with Sidak’s post hoc multiple-comparison tests. **, P<0.01, ***, *P*<0.001, ****, P<0.0001.

To investigate whether aging alters hepatic estrogen receptor signaling, we quantified the expression of estrogen receptors in livers from aging and young mice following hormone treatments. In aging mice, *Esr1* (ERα) remained the dominant estrogen receptor but was significantly reduced compared with young mice (Fig. 7A–B). In contrast, *Gper1* (GPER) expression was markedly elevated in aging mice and further increased by E_2_ treatment, whereas expression remained low in young mice regardless of hormone status (Fig. 7A, C). *Esr2* (ERβ) expression was detectable only at low levels and showed minimal regulation by hormone treatment in aging mice and was undetectable with qPCR in young mice (Fig. 7A). These findings demonstrate age-dependent estrogen receptor remodeling characterized by loss of ERα dominance and relative enrichment of GPER signaling.

Because constitutive GPER activation has been linked to NOX1-dependent reactive oxygen species production, we next assessed hepatic oxidative stress pathways. Expression of Nox4 was significantly increased in aging OVX+E_2_ mice compared with OVX controls and remained substantially higher than in young mice (Fig. 7D). These results are in line with previous observations that activation of ERα pathways attenuate NOX4-mediated reactive oxygen species (ROS) production in young adult mice^37^. Similarly, *Nfe2l2* (Nrf2), a master regulator of antioxidant defense, was elevated in aging sham and OVX+E_2_ mice, suggesting activation of compensatory antioxidant responses in the setting of increased oxidative stress (Fig. 7E). Notably, Nox1 expression was detectable only in aging sham and OVX+E_2_ mice and was undetectable in aging-OVX and all young groups (Fig. 7F), supporting activation of a GPER-NOX1 oxidative stress axis specifically in aging livers.

Consistent with increased oxidative stress, aging OVX+E_2_ mice exhibited marked induction of cellular stress-response genes. Gene expression of *Mycl* (bHLH transcription factor), *Cdk1* (Cyclin-dependent kinase 1), and *Eda2r* (Ectodysplasin A2 receptor) was significantly increased in aging OVX+E_2_ mice compared with both sham and OVX controls, whereas expression remained low and largely unresponsive to hormone treatment in young mice (Fig. 7G–I). *Cdk1* upregulation is consistent with activation of DNA damage repair pathways, while *Eda2r* has been associated with cellular senescence and stress signaling. Together, these findings suggest that aging and E_2_-treatment synergistically promote hepatic oxidative stress and cellular stress responses, potentially through age-dependent remodeling of estrogen receptor signaling toward increased GPER activity. This mechanism may contribute to the heightened inflammatory state and impaired cardiovascular benefits of hormone therapy observed in aging mice.

## Discussion

The present study investigated the mechanisms underlying the discordance between the favorable metabolic effects of menopausal hormone therapy and its inconsistent cardiovascular outcomes. Using mouse models of established atherosclerosis following surgical menopause, we demonstrate that although estrogen treatment consistently improves metabolic parameters during lipid normalization, its cardiovascular benefits are profoundly influenced by age. In aging mice, E_2_ treatment failed to reduce atherosclerotic burden despite improved lipid metabolism and insulin homeostasis. Instead, E_2_ promoted persistent systemic inflammation, impaired HDL function, increased plaque inflammation and vulnerability, and activated hepatic inflammatory and oxidative stress pathways. In contrast, young mice retained both the metabolic and cardiovascular benefits of estrogen treatment. Mechanistically, aging was associated with increased GPER expression, accompanied by increased NOX1 expression, oxidative stress and cellular stress signaling. Importantly, suppression of inflammation through *Ifnγ*^−/−^ bone marrow transplantation restored HDL function and reduced atherosclerosis during E_2_ treatment, highlighting inflammation as a critical determinant of residual ASCVD risk during hormone therapy.

The age-dependent effects observed in the present study closely parallel findings from major clinical trials of menopausal hormone therapy. Numerous studies, including HERS, WHI, ERA, WELL-HART, and WAVE, demonstrated that although hormone therapy improves several traditional cardiovascular risk factors, it fails to consistently reduce cardiovascular events in older women with established atherosclerosis. In contrast, the ELITE trial demonstrated that women initiating hormone therapy within six years of menopause experienced slower progression of carotid intima-media thickness, whereas no benefit was observed in women initiating therapy more than ten years after menopause^22^. Our aging mouse model recapitulates these clinical observations. Both young and aging mice exhibited improved metabolic parameters following E_2_ treatment; however, only young mice demonstrated reduced plaque inflammation, improved HDL function, and regression of atherosclerotic lesions. These findings suggest that the diminished cardiovascular efficacy of hormone therapy is not attributable to loss of estrogen responsiveness, but rather to age-dependent alterations in tissue responses to estrogen. Thus, aging fundamentally changes the biological consequences of estrogen signaling, uncoupling its metabolic benefits from cardiovascular protection.

One of the most important findings of the present study is the identification of age-associated remodeling of hepatic estrogen receptor signaling. In young animals, hepatic ERα remained the dominant estrogen receptor, consistent with previous studies demonstrating ERα-mediated regulation of lipid metabolism, insulin sensitivity, and mitochondrial function. The hepatic ERα dominancy in young mice also contributed to pronounced responsiveness to hormone treatments in liver lipid contents, given that estrogens limit liver fat accumulation by promoting fatty acid oxidation via hepatic ERα pathways^14,30^. In contrast, aging markedly reduced ERα expression while increasing hepatic GPER expression, suggesting a shift in estrogen signaling toward noncanonical pathways.

This age-associated receptor remodeling may contribute to activation of oxidative stress and DNA damage signaling in menopausal E_2_-treated aging mice. Constitutive activation of GPER has been shown to stimulate NOX1 activity and reactive oxygen species (ROS) production^38^. Consistent with this mechanism, Nox1 mRNA was induced exclusively in aging sham- and E_2_-treated mice but was undetectable in aging-OVX and all young groups, suggesting that aging and estrogen signaling cooperate to activate the GPER-NOX1 axis. Interestingly, Nox4 expression was generally higher in aging than in young mice but was reduced by E_2_ treatment compared with aging sham mice. Previous studies have reported that estrogens suppress Nox4 expression, likely through ERα signaling^39^. Despite this reduction in Nox4, aging E_2_-treated mice exhibited increased expression of multiple cellular stress-response genes, including *Cdk1*, *Mycl*, and *Eda2r*, together with induction of the antioxidant transcription factor Nrf2, indicating activation of compensatory antioxidant defense pathways in response to elevated oxidative stress. Collectively, these findings suggest that aging establishes a hepatic environment characterized by chronic oxidative stress, in which E_2_ treatment further amplifies ROS production through GPER-NOX1 signaling, leading to DNA damage responses and inflammatory activation.

Metabolic responses to estrogen remain preserved but are attenuated with aging. Despite the profound differences in cardiovascular outcomes, estrogen retained significant metabolic activity in both age groups. Lipid normalization substantially reduced plasma cholesterol and triglycerides in all mice, and E_2_ further improved triglyceride metabolism in aging animals while reducing fasting cholesterol and improving lipoprotein profiles in young mice. Likewise, hepatic lipid accumulation remained responsive to estrogen in both age groups. However, the magnitude of these metabolic responses was consistently greater in young mice. Young animals exhibited larger reductions in circulating lipids, greater improvements in lipoprotein composition, and more pronounced reductions in hepatic lipid accumulation by E_2_ treatment than aging mice. These findings suggest that hepatic responsiveness to estrogen progressively declines with aging. The marked reduction in hepatic ERα expression likely contributes to this diminished metabolic responsiveness, whereas increased GPER expression promotes oxidative stress and inflammatory signaling. In addition, hepatic ERβ expression was increased with aging. Although ERα is well recognized for its role in regulating lipid metabolism and glucose homeostasis, its anti-inflammatory and antioxidant functions in the liver remain less well defined. In contrast, ERβ exhibits a distinct cell type-specific distribution within the liver^40^. Whereas ERα is predominantly expressed in hepatocytes, ERβ is enriched in hepatic stellate cells, the principal fibrogenic cell population^41^. Previous studies have demonstrated that the anti-fibrotic actions of estrogen are mediated primarily through ERβ rather than ERα or GPER^41,42^. Therefore, the increased ERβ expression observed in aging mice may represent an adaptive response to age-associated oxidative stress and tissue remodeling, potentially reflecting activation or expansion of hepatic stellate cells. Although this hypothesis requires direct experimental validation, it is consistent with the increased expression of stress-response and tissue remodeling genes observed in our study. Collectively, these findings suggest that aging fundamentally remodels hepatic estrogen receptor signaling, shifting estrogen responses away from efficient metabolic regulation toward oxidative stress, inflammation, and tissue remodeling.

Our findings show that young mice preserve coordinated metabolic and cardiovascular protection. Unlike aging mice, young animals maintained coordinated improvements in both metabolic and cardiovascular outcomes during estrogen treatment. In addition to improved plasma lipid profiles, young mice demonstrated reduced circulating inflammatory cytokines, improved HDL antioxidant and cholesterol efflux capacity, decreased macrophage accumulation within plaques, and regression of established atherosclerotic lesions. Interestingly, several inflammatory genes remained estrogen responsive in young mice. However, their expression remained comparable to or below baseline levels and was not associated with increased systemic inflammation or plaque progression. These findings suggest that estrogen-induced inflammatory signaling may represent a normal physiological response that is effectively resolved in young animals but becomes maladaptive in aging tissues. Whether this difference reflects greater immune resilience, enhanced antioxidant capacity, preserved mitochondrial function, or improved resolution of inflammation remains unknown. Future studies employing single-cell transcriptomics and cell type-specific genetic models will be required to identify the hepatic and immune cell populations responsible for these age-dependent responses.

Inhibition of inflammation improves cardiovascular outcomes but also alters hepatic lipid metabolism. Our bone marrow transplantation experiments demonstrate that inflammation plays a causal role in limiting the cardiovascular benefits of estrogen therapy. Interestingly, suppression of inflammation also altered hepatic metabolic pathways. Although inflammatory and stress-response genes, including *Fgf21* and *Serpine1*, were substantially reduced following transplantation, expression of *Cers6* remained elevated. Based on our previous studies using liver-specific SHP knockout mice receiving *Ifnγ*^−/−^ bone marrow transplantation^27^, reduced inflammatory signaling was associated with decreased hepatic lipid utilization. We speculate that diminished lipid metabolism lowers metabolic stress, thereby reducing induction of adaptive stress mediators such as FGF21. However, decreased lipid turnover may also promote intracellular accumulation of lipid substrates that can be diverted toward ceramide synthesis, resulting in persistent upregulation of *Cers6*. These observations suggest that inflammatory signaling and hepatic lipid metabolism are tightly interconnected and may not always change in parallel. Future studies are needed to determine how inflammatory pathways coordinate hepatic lipid utilization, ceramide biosynthesis, and metabolic stress during hormone therapy.

In summary, the present study demonstrates that aging fundamentally alters the biological response to estrogen therapy. Rather than uniformly protecting against cardiovascular disease, estrogen promotes hepatic oxidative stress and inflammation in aging mice through age-associated estrogen receptor remodeling characterized by reduced ERα dominance and increased GPER signaling. Persistent inflammation impairs HDL function and limits regression of established atherosclerosis despite preserved improvements in systemic metabolism. Suppression of inflammation restores the cardiovascular benefits of estrogen, supporting inflammation-driven, non-lipid mechanisms as major contributors to residual ASCVD risk during hormone therapy. These findings provide a mechanistic insight into the age-dependent loss of cardiovascular benefits of hormone therapy and identify hepatic estrogen receptor remodeling, oxidative stress, and inflammatory signaling as promising therapeutic targets for improving cardiovascular outcomes in postmenopausal women receiving hormone therapy.

## Supplementary Material

Supplement 1

Supplement 2

## Figures and Tables

**Table 1.** Serum cytokine concentrations from young mouse groups.

(pg/ml)	Baseline	Sham	OVX	OVX+E_2_
**GM-CSF**	11±1.2	7.3±0.9^b^	6.6±0.3^b^	7.0±0.5^b^
**IFN_γ_**	5.9±1.0	3.9±0.2^b^	0.2±0.3^b, c^	0.2±0.4^b, c^
**IL-1B**	4.7±1.1	2.8±0.7^b^	1.5±0.2^b, c^	2.1±0.5^b^
**IL-4**	0.31±0.26	0.68±0.16^b^	0.16±0.12^c^	0.24±0.08^c^
**IL-6**	9.5±1.5	8.4±1.3	2.4±0.2^b, c^	4.1±1.4^b, c^
**IL-10**	2.8±0.5	9.3±1.3^b^	1.6±0.3^c^	2.6±0.6^c^
**KC**	320±140	362±56	656±154^b, c^	444±153
**MCP-1**	33±3.4	18±3.7^b^	30±1.1^c^	19±2.3^b^

All cytokines were measured by the Vanderbilt Hormone Assay and Analytical Service Core. GM-CSF: Granulocyte-Macrophage Colony-Stimulating Factor; KC: Keratinocyte chemoattractant; MCP-1: Monocyte Chemoattractant Protein-1.

b P<0.01 versus baseline

c P<0.01 versus the sham group.
