# Supplementary material for: Cardiovascular Benefits of Menopause Hormone Treatment is Age-Dependent
Source: bioRxiv. 2026 Jul 1:2026.06.29.734522. Preprint. [Version 1] doi: 10.64898/2026.06.29.734522 (PMC13345000; doi:10.64898/2026.06.29.734522)
Supplement: Supplement 1 [file media-1.pdf]

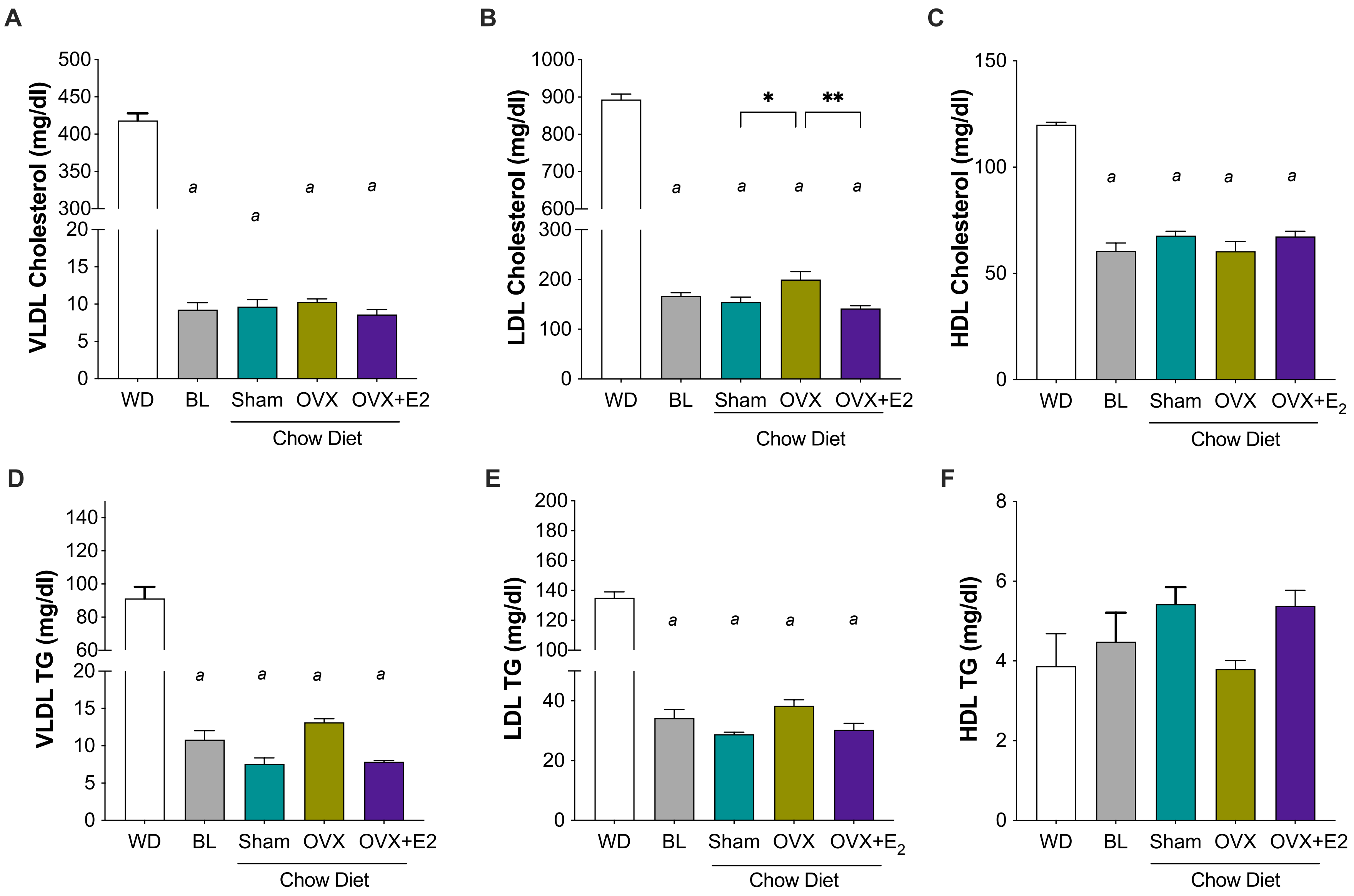

Suppl. Fig 1. Serum cholesterol and triglycerides (TG) distribution in lipoproteins from aging mice across hormone treatments were analyzed with FPLC. A-C: cholesterol distribution in VLDL (A), LDL (B), and HDL (C). D-F: TG distribution in VLDL (D), LDL (E), and HDL (F). FPLC were performed with serum samples pooled from 2-3 mice. Data are presented as mean ± SEM (n>=7). Statistical analyses were performed using one-way ANOVA. *a*, *P*<0.05 compared to Western diet (WD) conditions; *, P<0.05; **, P<0.01.

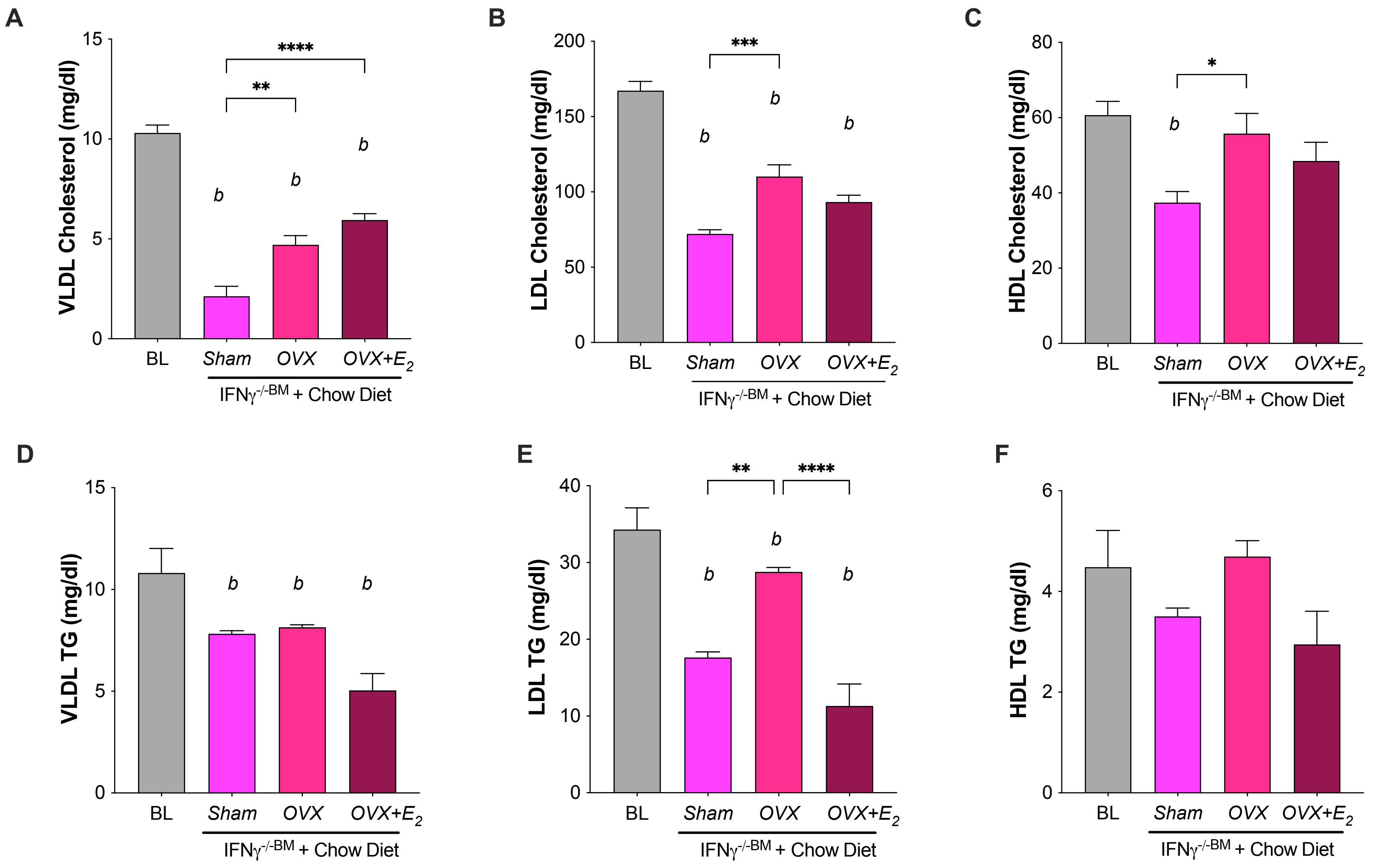

Suppl. Fig 2. Serum cholesterol and triglycerides (TG) distribution in lipoproteins from Ifnγ$^{-/-}$ recepient aging mice across hormone treatments were analyzed with FPLC. A-C: cholesterol distribution in VLDL (A), LDL (B), and HDL (C). D-F: TG distribution in VLDL (D), LDL (E), and HDL (F). FPLC were performed with serum samples pooled from 2-3 mice. Data are presented as mean ± SEM (n>=7). Statistical analyses were performed using one-way ANOVA. *b*, P<0.05 compared to baseline (BL) conditions. *, P<0.05, **, P<0.01, ***, P<0.001, ****, P<0.0001.

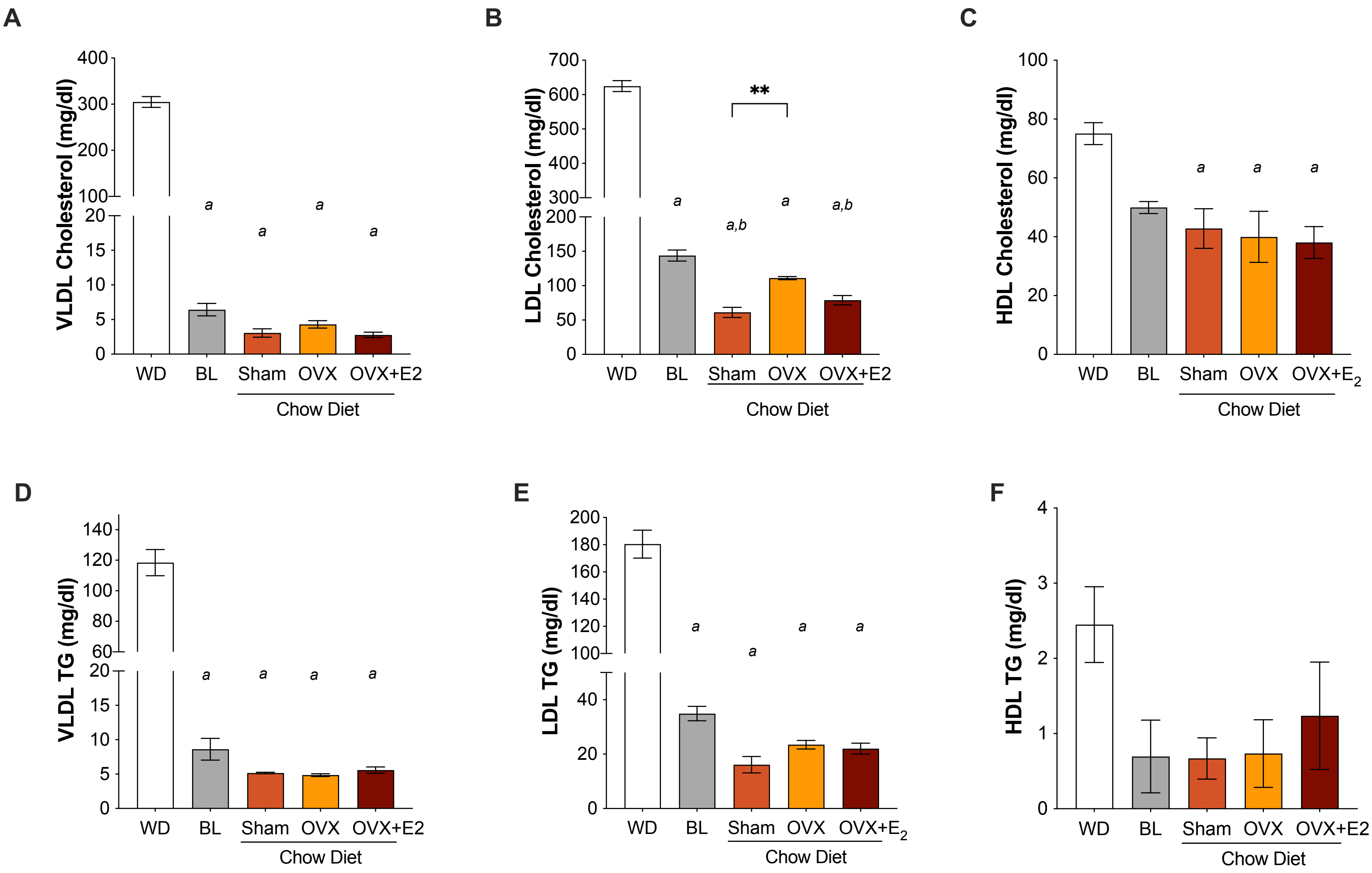

Suppl. Fig 3. Serum cholesterol and triglycerides (TG) distribution in lipoprotein fractions from young adult mice across hormone treatments were analyzed with FPLC. A-C: cholesterol distribution in VLDL (A), LDL (B), and HDL (C). D-F: TG distribution in VLDL (D), LDL (E), and HDL (F). FPLC were performed with serum samples pooled from 2-3 mice. Data are presented as mean ± SEM (n>=7). Statistical analyses were performed using one-way ANOVA. *a*, *P*<0.05 compared to Western diet (WD) conditions. *b*, *P*<0.05 compared to baseline (BL) conditions. **, P<0.01.

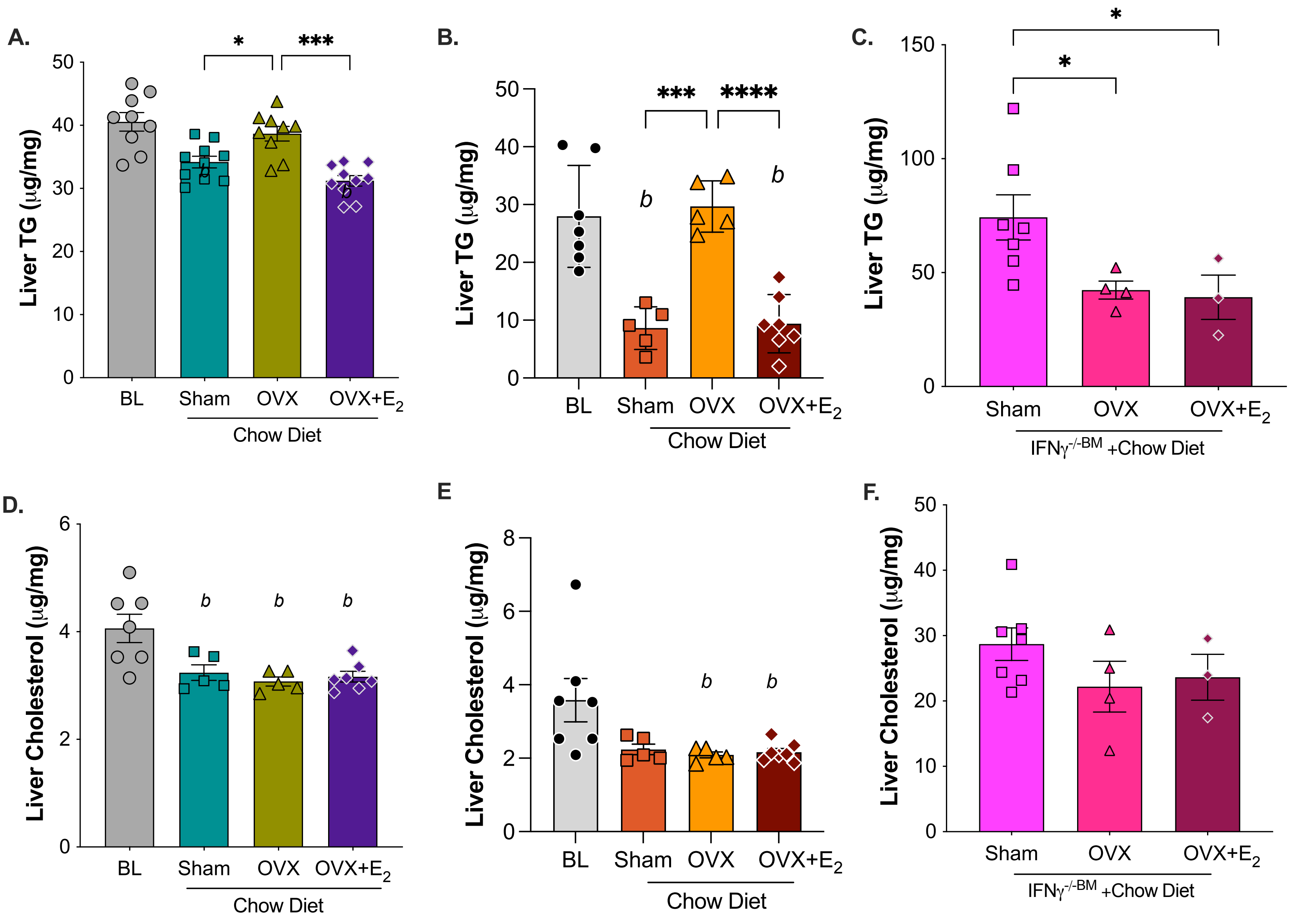

Suppl. Fig 4. Liver triglyceride (TG) and cholesterol contents from aging mice (A and D), young mice (B and E), and Ifnγ$^{-/-BM}$ recipient mice (C and F). Lipid levels for aging and young mice were determined using MS by the Vanderbilt Hormone Analysis Core lab. Lipid content for Ifnγ$^{-/-BM}$ recipient mice were measured after TCL separation of neutral lipids from liver lysates. Data are presented as mean ± SEM (n=5-7 for aging and young mice; n=3-7 for Ifnγ$^{-/-BM}$ recipient mice due to the limited amounts of liver tissues available). Statistical analyses were performed using one-way ANOVA. *b*, *P*<0.05 compared to baseline (BL) conditions. *, P<0.05; ***, P<0.001; ****, P<0.0001.

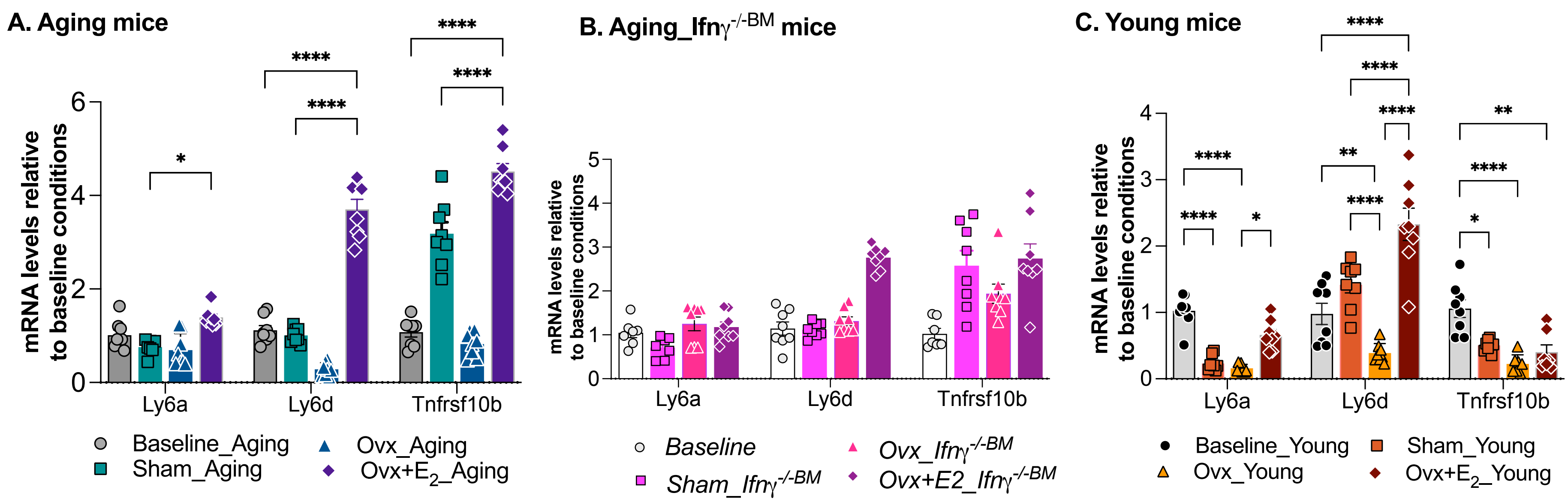

Suppl. Fig. 5. Aging- and estrogen-dependent regulation of hepatic inflammatory genes : Expression of genes associated with inflammation in aging mice (**A**), in aging mice receiving *Ifnγ*$^{-/-}$ bone marrow transplantation **(B)**, and young mice **(C).** Gene expression was determined by normalizing to 18s. Data are presented as fold change relative to the corresponding baseline groups. Data are shown as mean ± SEM (n=7-8). Statistical significance was determined by two-way ANOVA with Sidak's post hoc multiple-comparison tests. *, *P*<0.05, **, P<0.01, ****, P<0.0001.
