# Supplementary material for: Cardiovascular Benefits of Menopause Hormone Treatment is Age-Dependent
Source: bioRxiv. 2026 Jul 1:2026.06.29.734522. Preprint. [Version 1] doi: 10.64898/2026.06.29.734522 (PMC13345000; doi:10.64898/2026.06.29.734522)
Supplement: Supplement 2 [file NIHPP2026.06.29.734522v1-supplement-2.pdf]

bioRxiv preprint doi: https://doi.org/10.64898/2026.06.29.734522; this version posted July 1, 2026. The copyright holder for this preprint (which was not certified by peer review) is the author/funder, who has granted bioRxiv a license to display the preprint in perpetuity. It is made available under a CC-BY 4.0 International license.

Supplemental Table 1: TaqMan Assays used for qPCR

**Protein**	**Gene Symbol**	**Assay ID**
RNA, 18S Ribosomal 1	18S	Hs99999901_s1
S100 Calcium Binding Protein A8	S100a8	Mm00496696_g1
S100 Calcium Binding Protein A9	S100a9	Mm00656925_m1
Lymphocyte antigen 6 family member A	Ly6a	Mm00726565_s1
Lymphocyte antigen 6 family member D	Ly6d	Mm00521959_m1
Plasminogen activator inhibitor-1	Serpine1	Mm00435858_m1
TNF Receptor Superfamily Member 10b	Tnfrsf10b	Mm00457866_m1
C-X-C Motif Chemokine Ligand 9	Cxcl9	Mm00434946_m1
Signal Transducer and Activator of Transcription 1	Stat1	Mm01257286_m1
Ceramide Synthase 6	Cers6	Mm00556165_m1
Fibroblast Growth Factor 21	Fgf21	Mm00840165_g1
L-Myc, BHLH Transcription Factor	Mycl	Mm00493155_m1
Cyclin Dependent Kinase 1	Cdk1	Mm00772472_m1
Ectodysplasin A2 Receptor	Eda2r	Mm00723601_m1
Estrogen Receptor 1 (α)	Esr1	Mm00433149_m1
Estrogen Receptor 2 (β)	Esr2	Mm00599821_m1
G Protein-Coupled Estrogen Receptor 1	Gper1	Mm02620446_s1
NADPH Oxidase 1	Nox1	Mm00627696_m1
NADPH Oxidase 4	Nox4	Mm00549120_m1
